# Modelling land system evolution and dynamics of terrestrial carbon stocks in the Luanhe River Basin, China: a scenario analysis of trade-offs and synergies between sustainable development goals

**DOI:** 10.1007/s11625-021-01004-y

**Published:** 2021-07-16

**Authors:** Jiren Xu, Fabrice G. Renaud, Brian Barrett

**Affiliations:** 1 School of Interdisciplinary Studies, University of Glasgow, Dumfries, UK; 2 School of Geographical and Earth Sciences, University of Glasgow, Glasgow, UK

**Keywords:** Land system, Terrestrial carbon stocks, Luanhe River Basin, Sustainable development goals

## Abstract

A more holistic understanding of land use and land cover (LULC) will help minimise trade-offs and maximise synergies, and lead to improved future land use management strategies for the attainment of Sustainable Development Goals (SDGs). However, current assessments of future LULC changes rarely focus on the multiple demands for goods and services, which are related to the synergies and trade-offs between SDGs and their targets. In this study, the land system (combinations of land cover and land use intensity) evolution trajectories of the Luanhe River Basin (LRB), China, and major challenges that the LRB may face in 2030, were explored by applying the CLUMondo and InVEST models. The results indicate that the LRB is likely to experience agricultural intensification and urban growth under all four scenarios that were explored. The cropland intensity and the urban growth rate were much higher under the historical trend (*Trend*) scenario compared to those with more planning interventions (*Expansion*, *Sustainability*, and *Conservation* scenarios). Unless the forest area and biodiversity conservation targets are implemented (*Conservation* scenario), the forest areas are projected to decrease by 2030. The results indicate that water scarcity in the LRB is likely to increase under all scenarios, and the carbon storage will increase under the *Conservation* scenario but decrease under all other scenarios by 2030. Our methodological framework and findings can guide regional sustainable development in the LRB and other large river basins in China, and will be valuable for policy and planning purposes to the pursuance of SDGs at the sub-national scale.

**Supplementary Information:**

The online version contains supplementary material available at 10.1007/s11625-021-01004-y.

## Introduction

Natural resources, particularly land use and land cover (LULC) types and their associated changes, are central to sustainable development issues (Turner 1997). With continued societal and economic development, various human activities have significantly influenced the Earth’s surface to satisfy societal demand for food and living space, with considerable environmental consequences (Ellis et al. 2010; Malek et al. 2018), in which LULC is one of the most significant indicators of such impacts (Lambin and Meyfroidt 2011). LULC directly affects the status and integrity of ecosystems and their capacity to supply ecosystem services (Nelson et al. 2009). The United Nations General Assembly endorsed 17 sustainable development goals (SDGs) to assess and address the major challenges and threats of various social-ecological systems by 2030. These goals are related to ecosystem services (ES) and natural capital valuation (Barnaud et al. 2018; Costanza et al. 2014; Griggs et al. 2013), which are also affected by LULC. The 17 SDGs do not act individually or exist independently from each other, and some goals are thought to be mutually reinforcing or counter-acting (Nilsson et al. 2016a, b). The achievement of the SDGs crucially depends on maximising such synergies and resolving the existing trade-offs between various goals in different context strategies (Bowen et al. 2017; Kroll et al. 2019; Pradhan et al. 2017; Zhao et al. 2021). Most previous research addresses synergies and trade-offs between goals and between targets at the national scale, although such issues are also relevant at the sub-national scale (Hutton et al. 2018; Nerini et al. 2018; Scherer et al. 2018; Singh et al. 2018a). Therefore, a more holistic understanding of the complexities of future LULC associated with human demand for diverse ecosystem services and the potential trade-offs and synergies between SDGs is essential for providing a reference for decision making for sustainable development at the sub-national scale.

Because of complex driving factors and significant variations in LULC, it is challenging to predict LULC precisely (Verburg et al. 2004). LULC modelling is frequently utilised to understand and predict land system evolution trajectories considering socio-economic development and environmental protection policies at different scales (Alcamo and Schaldach 2006; Li et al. 2017b; Verburg et al. 2004, 2002). Many research groups have used simulation models to explore how future LULC changes would occur under various scenarios from a multidisciplinary perspective. Models such as the CLUE-S (Kucsicsa et al. 2019; Verburg and Overmars 2007), FORE-SCE (Rajib et al. 2016; Sohl et al. 2007), Markov chain and cellular automata analysis models (Hyandye and Martz 2017; Singh et al. 2018b) and agent-based models (Matthews et al. 2007; Parker et al. 2008) have been widely used to simulate LULC change at the local or river basin scales. Some large-scale land use models such as the LandSHIFT model (Alcamo and Schaldach 2006), GLOBIOM integrated assessment model (Ermolieva et al. 2015), and IMAGE model (Strengers et al. 2004) are used in regional or global LULC change simulations. These models typically simulate changes in mutually exclusive classes related to their land covers, such as forests, cropland, and built-up land. However, many land use changes do not directly affect the land cover at a location but instead relate to the land use intensity, while more nuanced changes between land use intensity and land management changes can have strong implications for ecological and environmental sustainability (Van Asselen and Verburg 2013). Yet, land uses can change in their extent but also in their intensity, thus allowing for multiple different change trajectories in response to an increase in demand. Moreover, most models are driven by demands for agricultural products and built-up areas, though the multiple non-material demands for afforestation and biodiversity protection will also affect the land system changes (DeFries and Rosenzweig 2010; Eitelberg et al. 2016). To overcome these constraints, a land system-based approach, which captures both land cover and land use at the landscape level, was proposed for land change modelling (Van Asselen and Verburg 2012). CLUMondo provides an innovative approach to simulate changes in land systems driven by various demands for commodities or services (Van Asselen and Verburg 2013). The integration of land management and land cover aspects allows the CLUMondo model to synchronously address multiple land system trajectories upon changes in driving factors. The CLUMondo model can not only allocate land system changes as determined by the regional demands of land cover types or different land use intensity, but also considers the local spatial preference, area restrictions, and competition between land systems. In CLUMondo, each good or service can be supplied by multiple land systems, and one land system can supply multiple goods or services simultaneously (Van Asselen and Verburg 2013).

In this study, we carry out scenario simulations based on the shared socio-economic pathways (SSPs) and regional policy and environmental conservation targets. The SSPs provide a framework for developing new socio-economic scenarios for global climate change studies and assessments of the broader sustainable development context (Ebi et al. 2014; O’Neill et al. 2014; Van Vuuren et al. 2014). The SSP framework is a critical component in the ongoing Intergovernmental Panel on Climate Change (IPCC) assessment of global climate change (O'Neill et al. 2016). The SSP framework has five scenarios. SSP1 is a sustainable pathway that is people-oriented and where land use is strongly regulated (Van Vuuren et al. 2017). International cooperation for climate change mitigation starts early (after 2020), and all land use emissions are priced at the level of carbon prices in the energy sector. SSP2 (Business-as-Usual) is a middle pathway between SSP1 and SSP3 that captures moderate challenges to mitigation and adaptation, with historically consistent trends in technological, economic and societal progress (Fricko et al. 2017). SSP3 is a regional rivalry pathway contrary to global cooperation. Countries focus on achieving energy and food security goals within their regions at the expense of broader-based development. Population growth is high in developing countries and low in industrialised countries. Environmental concerns remain a low international priority, resulting in substantial environmental degradation in some regions. Land use change is lightly regulated (Fujimori et al. 2017). SSP4 is a divided pathway in which inequality and stratification are increasing both across and within countries (Calvin et al. 2017). SSP5 is a fossil-fuelled development pathway in which the global economy grows rapidly, but people face severe mitigation challenges (Kriegler et al. 2017). Land use change is incompletely regulated, i.e. tropical deforestation continues, although at slowly declining rates over time. Crop yields are rapidly increasing. Unhealthy diets with high animal shares and high waste prevail (Popp et al. 2017).

As the most afforested river basin in North China and the main water source of Tianjin city, the fourth-largest urban area of China with a population of approximately 15 million, the Luanhe River Basin (LRB) plays a vital role in regional sustainable development (Yang et al. 2019). The LRB features marked afforestation over its upper basin since 1999, not only protecting Beijing from sandstorms originating from the Mongolian Plateau but also contributing significantly to soil and water conservation and carbon removal for climate change mitigation. To understand how the economic development, environmental protection and planning or policies will impact on future LULC and the attainment of the specify SDGs in the LRB, this study aims to analyse future LULC changes in the LRB to 2030 under different development scenarios that represent various socio-economic pathways and environmental conservation targets, and assess potential trade-offs and synergies based on these scenarios. The impact of these changes on the amount of carbon stored in the landscape is also calculated along with assessing the related implications for environmental management and planning purposes in pursuance of the SDGs.

## Materials and methods

### Study area

**Fig. 1:**
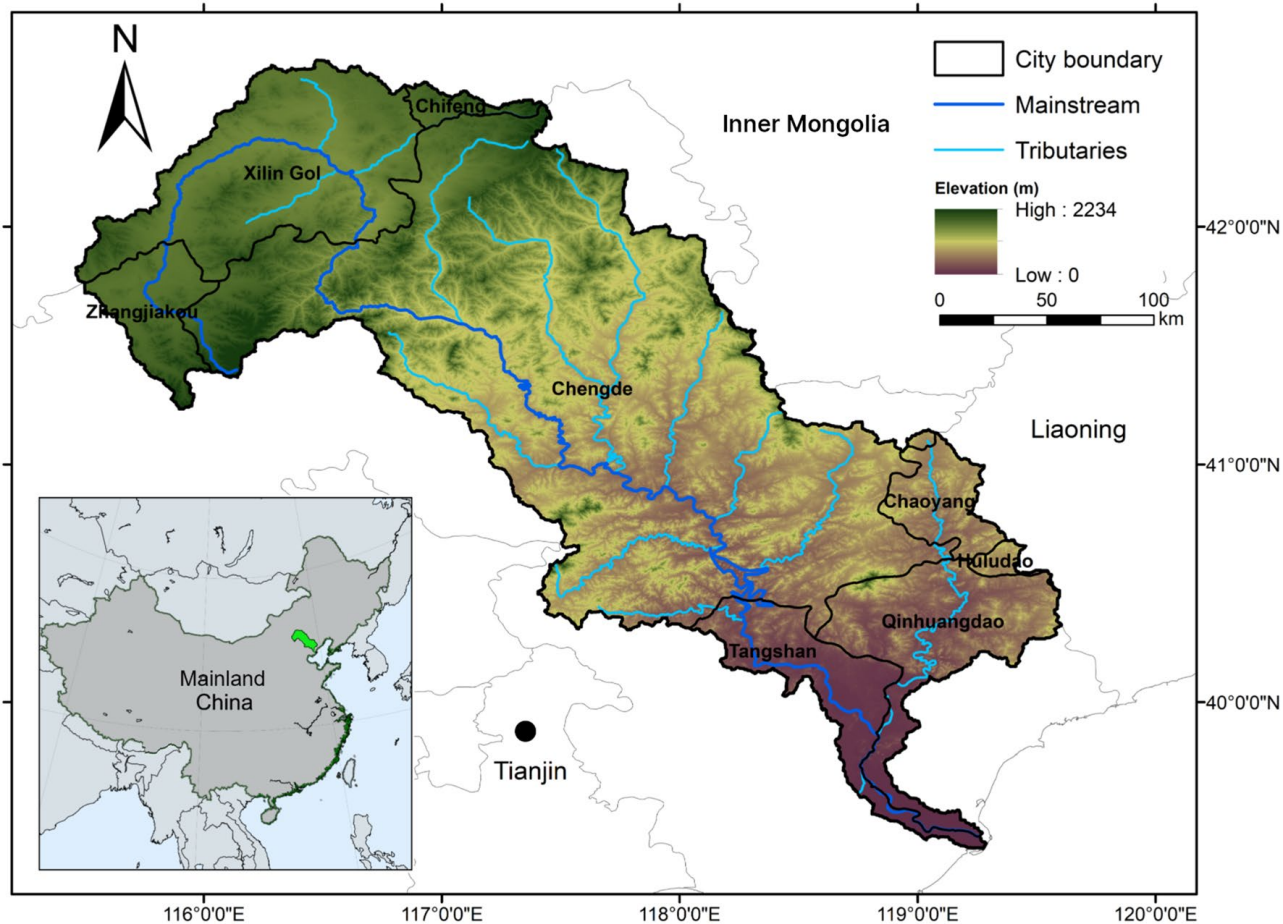
Location of the Luanhe River Basin. The mainstream of the Luanhe river is displayed in dark blue, and its associated tributaries in light blue

The LRB, covering a total area of approximately 45,000 km^2^, is located across a semi-arid area of North China (39°10′–42°30′ N, 115°30′–119°15′ E) (Fig. 1). It is an important ecological barrier to alleviate the effects of sandstorms from Mongolia Plateau and an important water resource for the Beijing–Tianjin–Hebei (BTH) region. The LRB is composed of three main types of terrain: plateau, mountainous area, and plain. The terrain inclines from northwest to southeast, and the average elevation is 766 m. It is situated in a typical temperate continental monsoonal zone with a semi-arid climate (Zhang et al. 2019), with an annual average temperature and precipitation during 1982–2015 of 7.0 ± 2.6 °C and 488.4 ± 80.7 mm, respectively (Wu et al. 2020). The temporal differences in precipitation distribution are significant, with heavy rainfall and relatively high temperatures in summer and less rainfall and lower temperatures in winter. The precipitation concentrates during the summer, which is about 200–550 mm, accounting for 66–76% of the annual precipitation (Geng et al. 2020). The upper reaches region is mainly covered by grassland, and the middle-lower reaches region is mainly covered by temperate forests, while the croplands and urban areas are located towards the eastern plains. The population in the LRB is approximately 5.4 million, with a density of 122 residents per km^2^ (Bi et al. 2018).

### CLUMondo framework

**Fig. 2:**
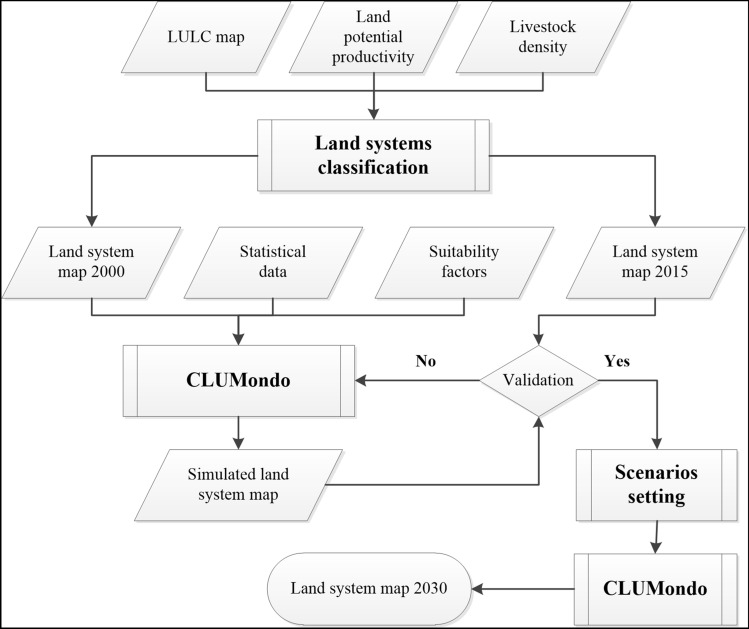
Land system change simulation workflow

The research was conducted in three steps. First, land systems of the LRB in the years 2000 and 2015 were mapped by integrating different datasets related to human–environment attributes. Then, the relationship between the land systems and local explanatory factors was calculated for the initial year (2000). Second, the CLUMondo model (Van Asselen and Verburg 2013) was parameterised and calibrated based on the 2015 land systems map. Finally, changes in the land systems from 2015 to 2030 were simulated under different scenarios, including alternative sets of demands for commodities and services and represented different pathways on managing LRB’s land resources. The overall approach is illustrated in Fig. 2.

#### Land system classification

The land system classification is based on three main classification factors: (1) land use and cover, (2) livestock, and (3) agricultural intensity. Land use/cover represents the landscape's composition, while livestock and agricultural intensity data represent important characteristics of land management and farming systems. Each variable's classification threshold was arbitrarily determined by the natural breaks in the variable distribution (van Asselen and Verburg 2012).

This study used the China National Land Use and Cover Change (CNLUCC) dataset (Liu et al. 2014; Xu et al. 2018b) from the Resources and Environmental Sciences Data Center of the Chinese Academy of Sciences. There are six land use and land cover categories, including farmland, forestland, grassland, water, urban land, and unused land in CNLUCC. The classification accuracy of CNLUCC was validated using nationwide field verification. Approximately, 10% of China's counties were randomly extracted, and all polygons in each county were validated through manual or visual interpretation to calculate the accuracy. The classification accuracies of selected polygons were more than 94.3% (Xu et al. 2018b).

**Fig. 3:**
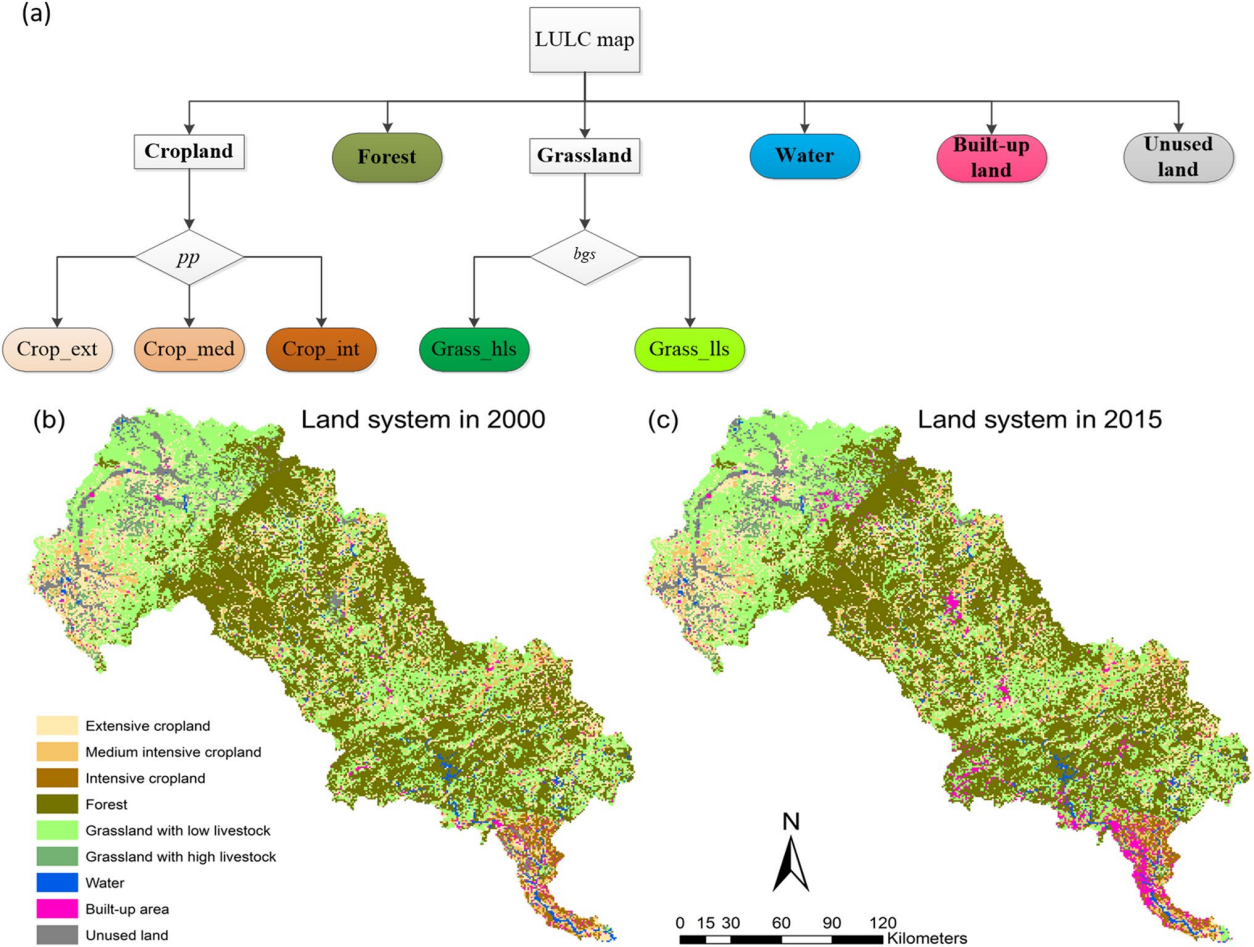
Land system classification approach. The italics represent the classification variables; the boldface represents the main land system categories; and colours represent the final land system classification outcome. Notes: pp is the potential cropland production, bgs is the bovines, goats and sheep density. Crop_ext is extensive cropland (pp < 0.4), Crop_med is medium intensive cropland (0.4 ≤ pp < 0.7), Crop_int is intensive cropland (pp ≥ 0.7), Grass_lls is grassland with low livestock (bgs < 100), Grass_hls is grassland with high livestock (bgs ≥ 100). Each variable's classification threshold was arbitrarily determined by the natural breaks in the variable distribution (van Asselen and Verburg 2012)

In this study, considering the data availability, we used the demand for crops, livestock, overall built-up land areas and overall forest areas as the drivers for the CLUMondo simulations. Similar to Liu et al. (2017), the land system of the forest, water, built-up land and unused land were firstly delineated using previously processed LULC data. In the LRB, crops are produced by cropland, and livestock (bovines, goats and sheep) are produced by grasslands, therefore the potential cropland production and livestock density were used to classify cropland and grassland systems, respectively. The dataset used for land system delineation is shown in Table 1. The specific classification procedure and delineated land system maps for 2000 and 2015 are illustrated in Fig. 3.

**Table 1:** Data used for land system delineation and the explanatory variables

Data application	Main category	Factors	Unit	Source
Delineate Land systems	Land use/cover data		Class	Resources and Environmental Sciences Data Center, Chinese Academy of Sciences (RESDC, http://www.resdc.cn/)
	Potential cropland production		Index (0–1) normalized from tons/km^2^	
	Livestock density		heads/km^2^	Global Distribution of Livestock (Robinson et al. 2014)
Explanatory factors	Climatic	Mean annual temperature	℃	RESDC (http://www.resdc.cn/)
		Annual Precipitation	Mm	
		≥ 10℃ Accumulated temperature	℃	
		Moisture index	%	
	Topographic features	Altitude	m	NASA SRTM V3.0
		Slope	degree	derived from Altitude
		Landforms	–	RESDC (http://www.resdc.cn/)
	Soil characteristic	Sand content	%	HWSD v1.2 (http://www.fao.org/soils-portal/soil-survey/soil-maps-and-databases/harmonized-world-soil-database-v12/en/)
		Silt content	%	
		Clay content	%	
		Organic content	%	
		pH	−log(H^+^)	
		Drainage	Class	
		Soil type	–	
	Socioeconomic	Market influence	USD/person	Verburg et al. (2011)
		Market accessibility	Index (0–1)	
		Population density	People/km^2^	RESDC (http://www.resdc.cn/)
		GDP	USD/km^2^	
Policy				
	Future demand	Crop production	Ton	National planning on medium- and long-term food security (National Development and Reform Commission of China 2008)
		Livestock	Head	
		Built-up area	km^2^	General Land Use Planning in Hebei Province (2006–2020) (Hebei Provincial Department of Land and Resources 2010)
		Forest area	km^2^	National Forest Management Planning (2016–2050) (State Forestry Administration of China 2016), Land greening planning of Hebei Province (2018–2035) (Hebei Provincial Department of Natural Resources 2018), Implementation plan of afforestation in Zhangjiakou city and Chengde Bashang area of Hebei Province (State Forestry Administration of China 2019)

#### Parameterisation of the CLUMondo Model

**Fig. 4:**
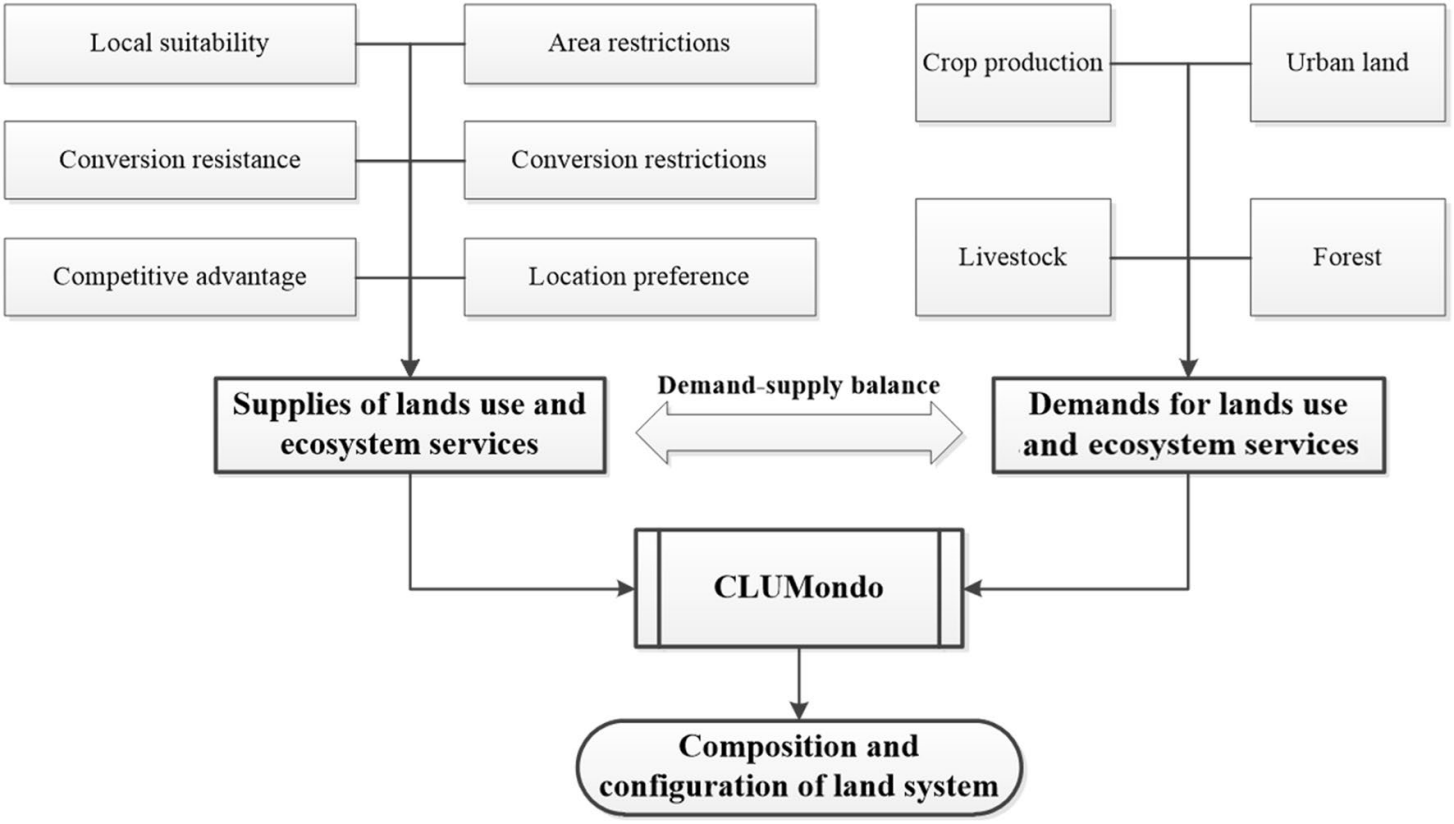
Main concept and workflow of CLUMondo model (Adapted from Van Asselen and Verburg, 2013)

CLUMondo is a forward-looking model that is specifically designed to simulate changes in land cover and changes in land use intensity. Also, it can represent multifunctional land. At the core, the simulation of land use change is based on an empirical analysis of location suitability combined with the dynamic simulation of competition and interactions between the spatial and temporal dynamics of land use systems. The basic principle of the CLUMondo model is to determine the spatial allocation of land systems through ensuring that the allocated land systems fulfil the regional demands for ecosystem goods and services in consideration of the regular supply of these goods and services by the different land systems (Van Asselen and Verburg 2013) (Fig. 4). The CLUMondo model is subdivided into two distinct modules: a non-spatial demand module and a spatially explicit allocation module. The non-spatial module indicates the changes in demand at the level of the entire model region. In this case, demands can refer to the demand for an area of specific land use and a number of goods or services. In the allocation module, these demands are subsequently translated into land use changes at specific locations in the study area, using a raster-based system. In CLUMondo, demands are external input to the system, while the allocation is determined by the model’s allocation algorithm, which is supported by the user interface. Land use demands can be derived in different forms, ranging from simple trend extrapolations to complex economic models. More information about the CLUMondo model and parameter settings can be found in Van Asselen and Verburg (2013).

The demands considered in our study included crop production, livestock, urban land and forest areas. To meet these demands, the land systems are allocated according to their capacity to supply these demands and the local biophysical and socio-economic conditions. To explore the extent to which land systems' spatial determinants can be explained by the independent factors, 18 biophysical and socio-economic factors, including climate, soil properties, topography, and vegetation type, were used (Table 1). Throughout the research period, the driving factors' stability was considered along with the availability, consistency, and quantifiability of the data. Detailed information related to the explanatory factors is also listed in Table 1. To avoid errors caused by data upscaling or downscaling, all data were resampled into a consistent spatial coordinate system (GCS_Krasovsky_1940, Albers Conical Equal Area) and resolution (1000 × 1000 m), the same spatial reference as the delineated land systems map. The number of explanatory factors was reduced to 12 after multiple collinearity assessments, wherein any two factors with a correlation coefficient > 0.7 were excluded. A structured sampling method was then applied to minimise spatial autocorrelation and balance the sample grid, and binominal logistic regressions were performed between the sampled location factors and specific land system (Castella et al. 2007; Ornetsmüller et al. 2016; van Asselen and Verburg 2012). The location factor is shown to calibrate the predicted land use by the information on the area under the curve (AUC), representing the accuracy between 0 and 1 of the calculated regression. Table 2 shows the results of the suitability analysis and the AUC value of each land system. The AUC values for all land systems exceeded 0.69, indicating that selected factors could reasonably explain land systems' spatial distribution.

**Table 2:** Suitability analysis and the AUC value of each land system

	Climactic		Topographic features	Soil parameters						Socioeconomic			
Land System	Temperature (℃)	Moisture (%)	Landforms	Sand content (%)	Clay content (%)	Organic content (%)	pH (−log(H +))	Drainage (Class)	Soil type	Market influence (USD/person)	Population density (people/km^2^)	GDP (USD/km^2^)	AUC
Crop_ext	+	−		−	−				+		+	−	0.69
Crop_med	+	−	+						+		+		0.73
Crop_int	+	−				+							0.95
Forest	+	+		−			−	+					0.78
Grass_lls				+			+	+	+	−	−	+	0.74
Grass_hls			−	−				+	+				0.69
Water				+				−	+				0.76
Built-up		−							+	+	+		0.81
Unused land					+				+		−	+	0.83

The “ + ” means a positive correlation between the explanatory factor and land system, while the “−” means a negative correlation between the explanatory factor and land system

A land system lookup table indicating the resistance factor, relative order of the land system’s contribution to fulfilling a specific demand, and their capacity to supply this demand was developed (see Table 3). The resistance factor is one of the land system type-specific settings that determine the temporal dynamics of the simulation, which is related to the reversibility of land use changes. Land system types with high capital investment or irreversible impact on the environment will not easily be converted into other uses as long as there are land requirements for those land systems. The land system with a higher value (≥ 0) has a higher-order for fulfilling the ecosystem services. The crop production provided by cropland systems was calculated based on potential cropland production from the Resources and Environmental Sciences Data Center, Chinese Academy of Sciences (http://www.resdc.cn/), and the livestock number carried by grassland with livestock systems was calculated based on Global Distribution of Livestock (Robinson et al. 2014). Land system requirements of the LRB for supplying the demands between 2000 and 2015 were calculated by extrapolating trends (Kurniawan 2014; Verburg and Veldkamp 2004). Additionally, a conversion matrix was used to indicate which land system conversions were possible. The conversion elasticity is derived from referring to previous studies (Jin et al. 2019; Liu et al. 2017; Lu et al. 2009; Park et al. 2011; Zhou et al. 2013) and from repeatedly modifying the calibration of the model to achieve the optimal simulating effect.

**Table 3:** The resistance factors and lookup values for each land system

Land system	Resistance factor	Crop production (Tons/pixel)		Livestock (Heads/pixel)		Urban land (km^2^/pixel)		Forest (km^2^/pixel)	
		Conversion order		Conversion order		Conversion order		Conversion order	
Crop_ext	0.6	1	212.2	0	0	0	0	0	0
Crop_med	0.7	2	456.3	0	0	0	0	0	0
Crop_int	0.8	3	723.7	0	0	0	0	0	0
Forest	0.8	0	0	0	0	0	0	1	1
Grass_lls	0.5	0	0	1	46.6	0	0	0	0
Grass_hls	0.6	0	0	2	124.6	0	0	0	0
Water	0.9	0	0	0	0	0	0	0	0
Built-up	0.9	0	0	0	0	1	1	0	0
Unused land	0.8	0	0	0	0	0	0	0	0

The resistance factor is one of the land system type-specific settings that determine the temporal dynamics of the simulation, which is related to the reversibility of land use changes

#### Model validation

Based on the two delineated land system maps, we simulated the land system changes in the LRB from 2000 to 2015. The model's accuracy was assessed by validating the simulation results using the actual land system map in 2015. Three indicators, including *Kappa simulation*, *Kappa transition*, and *Kappa transition location,* were used to assess the accuracy. *Kappa simulation* (− 1 to 1) is the coefficient of agreement between the simulated land use transitions and the actual land use transitions. *Kappa transition* (0 to1) expresses the agreement in the quantity of land use transitions, and *Kappa transition location* (− 1 to 1) expresses the degree to which the transitions agree in their allocations. These indices measured the consistency between the simulation results and actual land systems from different aspects, which have been successfully used in evaluating the CLUMondo simulated results (Jin et al. 2019; Liu et al. 2017). Further details regarding calculations and the indices used can be found in van Vliet et al. (2011).

### Scenario formulation

Four scenarios: *Trend*, *Expansion*, *Sustainability*, and *Conservation* were designed based on different socio-economic development and environmental protection targets, local plans and policies, and the information from a stakeholders’ workshop, to explore land system evolution trajectories of the LRB and major challenges that the river basin may face in the future. A stakeholder workshop was held in Tianjin, China, in October 2019 to collate information on the major challenges and their drivers in the basin, local policies, future development plan, and views on ecosystem services in the LRB. 15 stakeholders with extensive theoretical and practical knowledge of the local environment from government bodies, research institutes, and companies participated in the workshop. Full ethical clearance was granted by the University of Glasgow`s research ethics committee for the stakeholder consultation. The study period was selected as 2015–2030 to ensure temporal consistency of different datasets and align with the SDG targets achievement date.

#### Scenario 1: Trend

The *Trend* scenario follows the middle-of-the-road shared socio-economic pathway (SSP2), a pathway of the socio-economic trend that does not shift markedly from historical patterns, with relatively low commitment to achieve development goals (O’Neill et al. 2014). In CLUMondo, this scenario is driven by the demand for crop production, livestock, and built-up areas. The average annual change rates of demand for crop production and livestock were calculated using the data from the Statistical yearbook of Hebei Province (2015) (Hebei provincial bureau of statistics 2016). The annual demand for the built-up area was calculated based on land system change between 2000 and 2015. Demands for crop production, livestock number, and built-up space were predicted for the next 15 years using the projected change rates.

#### Scenario 2: Expansion

The *Expansion* scenario follows the fossil-fuelled development shared socio-economic pathway (SSP5), where people exploit abundant fossil fuel resources, the global economy grows at the highest speed, and the global urbanisation rate reaches 92% in 2100. The SSP5 scenarios mark the upper end of the scenario literature in fossil fuel use, food demand, energy use and greenhouse gas emissions. The annual change rates for the demand of the built-up area were derived from the future built-up land expansion in China under SSP5 (Chen et al. 2020), and the annual change rates for demands of crop and livestock were derived from the SSP database (https://tntcat.iiasa.ac.at/SspDb).

#### Scenario 3: Sustainability

The *Sustainability* scenario follows the sustainable shared socio-economic pathway (SSP1). The annual demand for crop production and livestock was derived from the projected annual change rates stated in “National planning on medium- and long-term food security” (National Development and Reform Commission of China 2008). The built-up area's annual demand was calculated based on General Land Use Planning in Hebei Province (2006–2020) (Hebei Provincial Department of Land and Resources 2010). It should be noted that the meaning of “*Sustainability*” used for describing the scenario here is not completely consistent with the concept of sustainability in SDGs. The “*Sustainability*” scenario follows the “sustainable” shared socio-economic pathway (SSP1), of which the demand for crop production, livestock and build-up area were derived from the relatively sustainable national or regional planning.

#### Scenario 4: Conservation

The socio-economic context of the *Sustainability* scenario was used as a baseline for the *Conservation* scenario and extended by the implementation of the ecological restoration and protection policy targets. According to the stakeholders and the policy review, a series of policies promoting afforestation have been implemented since 2015 in the Luanhe River Basin for biodiversity conservation and sand fixation. Based on planning data published in the National Forest Management Planning (2016–2050) (State Forestry Administration of China 2016), Land greening planning of Hebei Province (2018–2035) (Hebei Provincial Department of Natural Resources 2018) and Implementation plan of afforestation in Zhangjiakou city and Chengde Bashang area of Hebei Province (State Forestry Administration of China 2019), demands for forest area were linearly interpolated to obtain yearly demand quantities. Demand model parameters related to the four scenarios are described in Table 4.

**Table 4:** Average annual percentage change in demand from 2015 to 2030 for the different scenarios

Demand	Trend	Expansion	Sustainability	Conservation
Crop production (Ton)	2.1%	1.5%	1%	1%
Livestock numbers (Head)	2.7%	3.9%	0.9%	0.9%
Built-up land (km^2^)	9.6%	1.9%	0.7%	0.7%
Forest (km^2^)	n/a	n/a	n/a	0.4%

n/a refers to the demand which is not specified in the scenario formulation but will be allocated by the CLUMondo model

### Terrestrial carbon storage

The InVEST carbon storage module uses a simplified carbon cycle that quantifies the amount of static carbon storage and dynamic sequestration or loss based on four basic carbon density pools: aboveground biomass (*c_above*), belowground biomass (*c_below*), soil (*c_soil*), and dead organic matter (*c_dead*) (Sharp et al. 2020). The carbon in each pool was then aggregated over different land use types to estimate carbon storage across the landscape. The carbon storage ${S}_{m,i,j}$ for a given grid cell $(i,j)$ with land use type $m$ can be calculated as:

1 $${C}_{m,i,j}=A\times \left({c\_above}_{m,i,j}+{c\_below}_{m,i,j}+{c\_soil}_{m,i,j}+ {c\_dead}_{m,i,j}\right),$$

where $A$ is the actual area of each grid cell (ha) and ${c\_above}_{m,i,j}, {c\_below}_{m,i,j}, {c\_soil}_{m,i,j}$ and ${c\_dead}_{m,i,j}$ are the aboveground carbon density (MgC∙ha^−1^), belowground carbon density (MgC∙ha^−1^), soil organic carbon density (MgC∙ha^−1^), and dead organic matter carbon density (MgC∙ha^−1^) for grid cell $(i,j)$ with land use type *m*. Hence, carbon storage *C* across the whole region can be calculated as:

2 $$\mathrm{C}= \sum_{m=1}^{n}{C}_{m,i,j}.$$

Since currently available data of carbon pools are all estimated based on the land use and land cover (LULC) types rather than land system types; in this research, the projected future land system types were regrouped into land use and cover types. The terrestrial carbon storages in the LRB in the baseline and under different future scenarios were calculated based on the carbon storage in cropland (including extensive cropland, medium intensive cropland, and intensive cropland), forest, grassland (including grassland with high livestock and grassland with low livestock), built-up land, and unused land. The four carbon pools in each land use and cover types were collected by a literature review (Table 5). For maximising the carbon pool data gathered from various secondary sources within the study area: (1) we gave priority to the measured data for the LRB. For example, the *c_above*, *c_below*, *c_soil* and *c_dead* in the forest were derived from the measured data of mean carbon densities in the forest of the LRB by Wang and Gao (2009). Most of the grassland in the LRB is located in Inner Mongolia. Therefore, the *c_soil* in grassland was derived from the measurement data of soil organic carbon density in Inner Mongolia’s grassland by Jin et al. (2018); (2) For the carbon pools of which measured data for LRB was not available, we used the measured data for Hebei province. For example, the built-up land and unused land, the *c_soil* wAS derived from the measured data for Hebei province by by Xi et al. (2010); (3) For the rest of the carbon pools, we used the data derived from the national mean carbon densities in China by Tang et al. (2018) and Fang et al. (1996). It should be noted that in the InVEST carbon storage module, the carbon density of each terrestrial pool was assumed not to have changed during the modelling period.

**Table 5:** Carbon pools of different LULC types in InVEST (units: MgC·ha^−1^)

*LULC type*	*c_above*	*c_below*	*c_soil*	*c_dead*	*Sources*
Cropland	2.66	0.4	92.04	0	Tang et al. (2018)
Forest	27.58	4.96	157.14	8.24	Wang and Gao (2009)
Grassland	0.43	4.4	81.2	0.08	Jin et al. (2018); Tang et al. (2018)
Built-up land	0	0	78	0	Xi et al. (2010)
Unused land	0.1	0	72.4	0	Fang et al. (1996); Xi et al. (2010)

*c_above* refers to the aboveground biomass. *c_below* refers to the belowground biomass. *c_soil* refers to soil organic carbon. *c_dead* refers to the dead organic matter

## Results

### Land system changes from 2015 to 2030 under different scenarios

**Fig.5:**
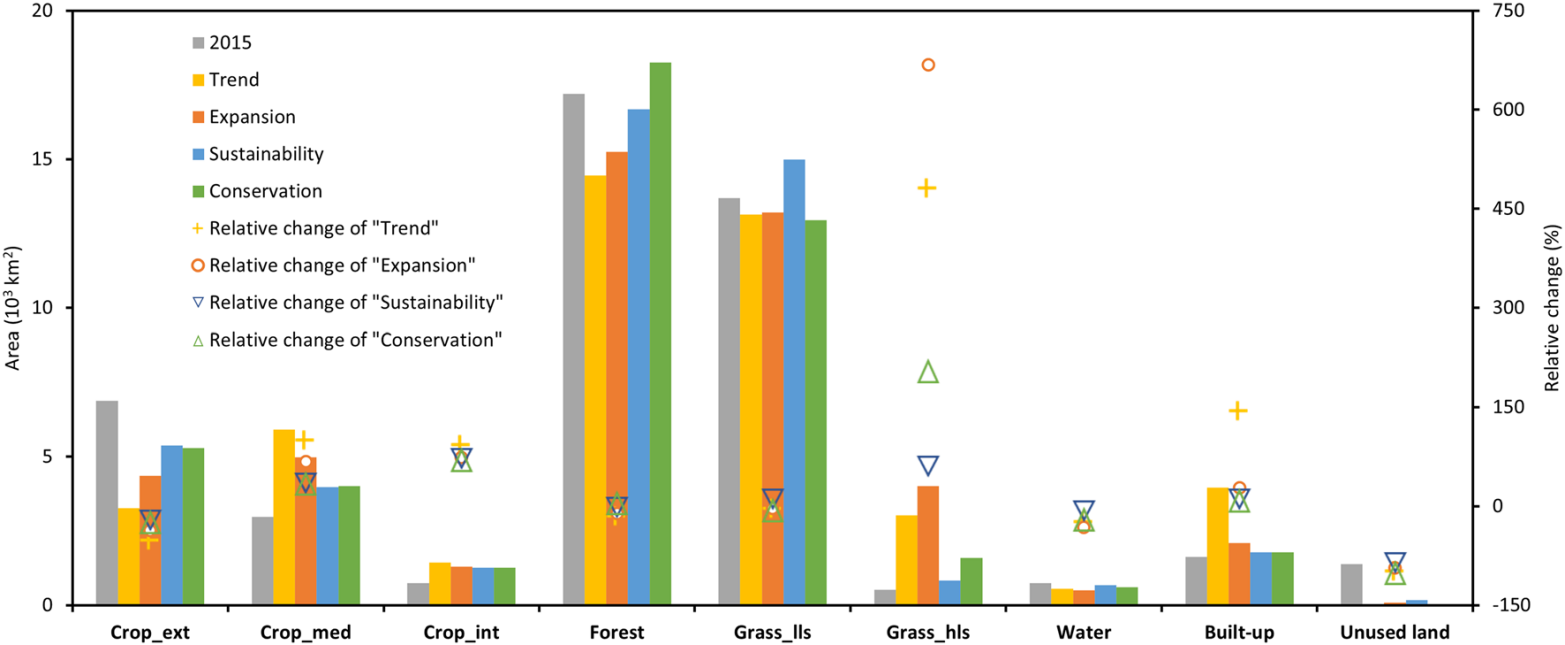
Absolute and relative changes in land system areas in 2015 and predicted values under various scenarios for 2030

**Fig.6:**
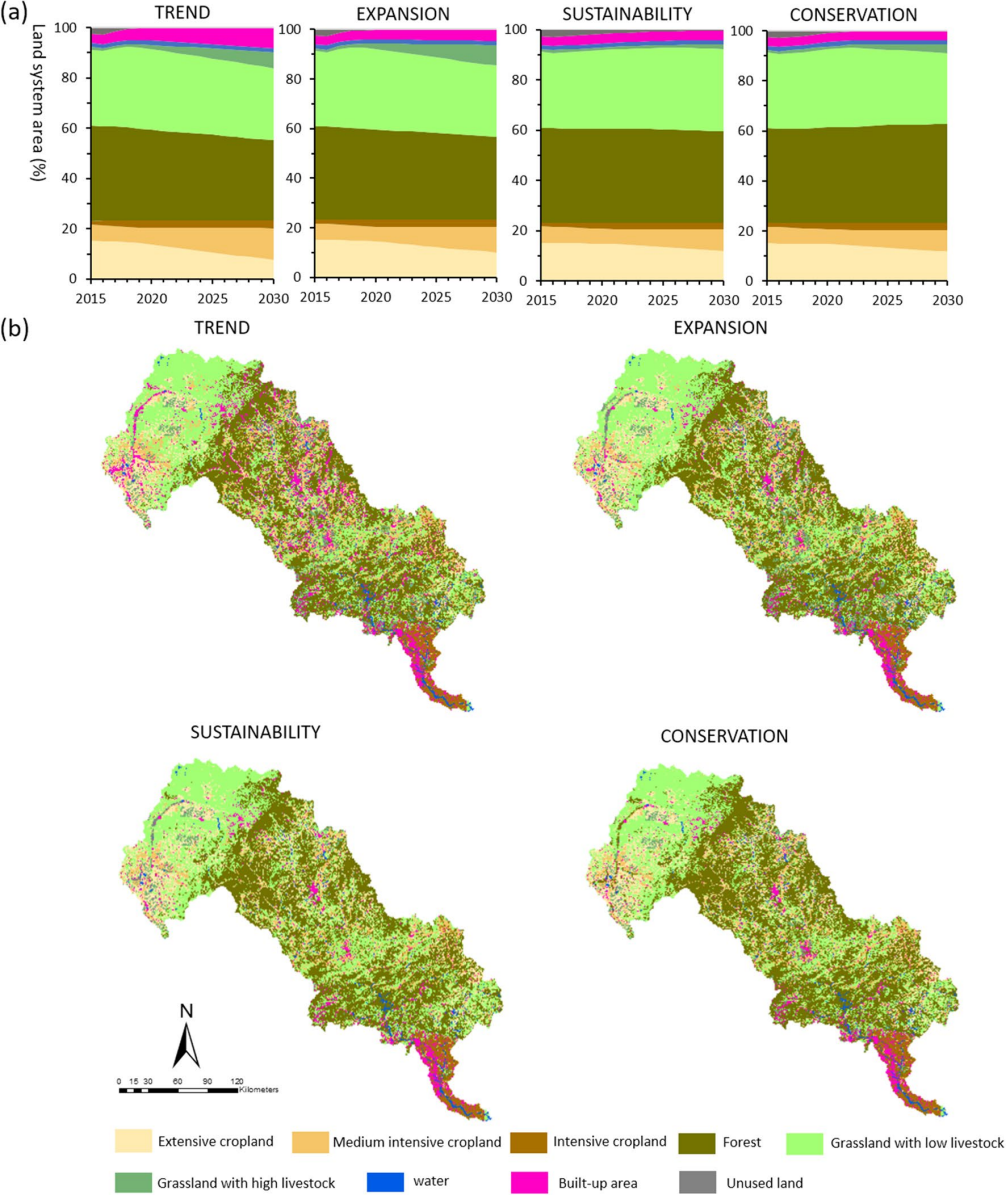
Land system change from 2015 to 2030 in the LRB under different scenarios. (**a**) percentage of land system area (%). (**b**) Land system maps in 2030

The interplay between demands, spatial policies and competition for the nine land system types led to different land system change trajectories from 2015 to 2030 under the *Trend*, *Expansion*, *Sustainability* and *Conservation* scenarios (Table 6, Tables S1–S4, Figs. 5 and 6). The cropland systems transformed into more intensive versions (Crop_med and Crop_int) in all four scenarios and all predict a decline in extensive cropland. The *Trend* scenario experienced the highest amount of cropland intensification due to its higher demand for crop production, with a 111% increase of intensive croplands, a 94% increase of medium intensive croplands and a 50% decrease of extensive cropland in the LRB from 2015 to 2030 (Table 6). Although built-up areas accounted for a small portion of the LRB, they exhibited a clear increasing trend in all four scenarios. The forest areas are projected to decrease in the *Trend*, *Expansion*, and *Sustainability* scenarios. Simultaneously, they recorded slight increases in the *Conservation* scenario because of the forest and biodiversity conservation targets. A considerable portion of the grassland intensification was related to increased demand for livestock. The most significant increase was observed in the *Expansion* scenario, where large areas of grassland systems with low-density livestock and unused land were replaced by grassland systems with high-density livestock. The *Sustainability* and *Conservation* scenarios presented a relatively smaller increase in the grassland systems with high-density livestock compared to the other two scenarios. This can be attributed to sustainable development planning and targets, restricting unreasonable development and unreasonable environmental resources utilisation. The areas of water and unused land were projected to be decreased under all scenarios in 2030. Most of these decreased water areas were projected to be replaced by grassland systems with low-density livestock. The most significant decrease of the unused land area was projected in the *Conservation* scenario, and most of these decreased unused lands were projected to be explored for forests and grassland systems with low-density livestock.

**Table 6:** Change in the land system from 2015 to 2030 in the LRB under four different scenarios

Land system	Area percent in 2015 (%)	Area percent in 2030 (%)				Change area from 2015 to 2030 (10^3^ km^2^)			
		Trend	Expansion	Sustainability	Conservation	Trend	Expansion	Sustainability	Conservation
Extensive cropland	15.12	7.15	9.51	11.74	11.55	− 3.61	− 2.53	− 1.51	− 1.60
Medium intensive cropland	6.50	12.92	10.87	8.70	8.78	2.95	2.01	1.01	1.05
Intensive cropland	1.62	3.12	2.82	2.77	2.78	0.69	0.55	0.53	0.53
Forest	37.59	31.58	33.32	36.43	39.88	− 2.75	− 1.96	− 0.53	1.05
Grassland with low livestock	29.94	28.70	28.86	32.76	28.30	− 0.56	− 0.49	1.30	− 0.74
Grassland with high livestock	1.14	6.61	8.76	1.82	3.48	2.51	3.49	0.31	1.07
Water	1.63	1.23	1.11	1.49	1.32	− 0.18	− 0.24	− 0.06	− 0.14
Built-up	3.44	8.67	4.57	3.91	3.91	2.34	0.46	0.16	0.16
Unused land	3.03	0.02	0.19	0.39	0.00	− 1.37	− 1.29	− 1.20	− 1.38

### Simulation of terrestrial carbon storage from 2015 to 2030

**Fig.7:**
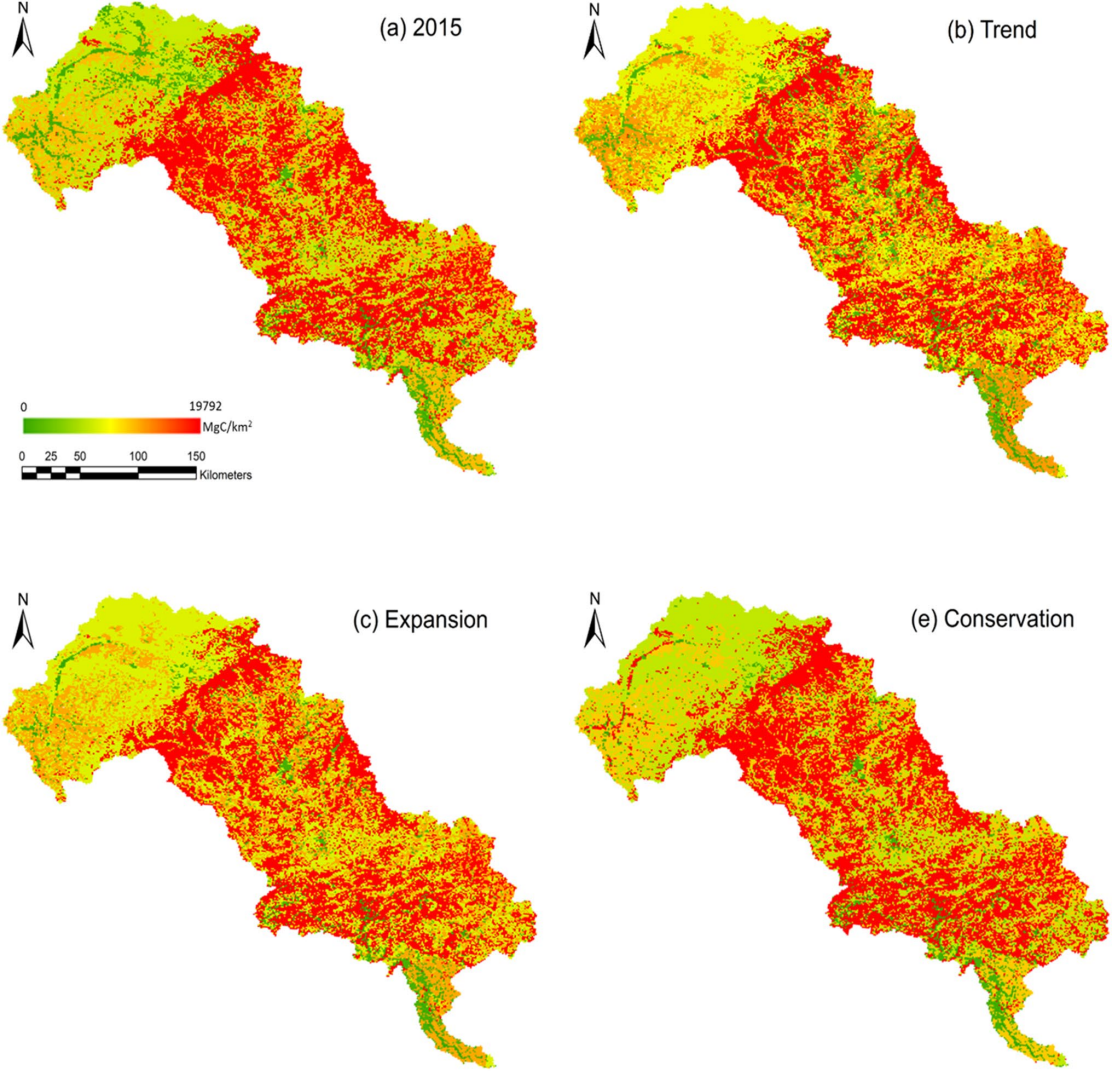
Spatial distribution of terrestrial carbon storage of the LRB in 2015 and 2030 under four different scenarios

We estimated potential regional carbon storage and change under the four different scenarios from 2015 to 2030 in the LRB (Table 7 and Fig. 7). The largest carbon pool is in the forests, followed by the grasslands, croplands, built-up lands, and unused lands. The results show that unless the biodiversity conservation targets are implemented (*Conservation* scenarios), carbon storage manifests a decreasing tendency from 2015 to 2030 under all the other three scenarios. However, the carbon losses would significantly reduce under the *Sustainability* scenario following the sustainable shared socio-economic pathway. The terrestrial carbon storage will decrease by 29.2 MtC, 18.4 MtC and 3.9 MtC under the *Trend*, *Expansion* and *Sustainability* scenarios, and increase by 14.6 MtC under the *Conservation* scenario. The change of soil carbon storage (*c_soil*) is the main driver of total terrestrial carbon storage change from 2015 to 2030, followed by the aboveground carbon pool (*c_above*).

**Table 7:** Terrestrial carbon storage (MtC) of the LRB in 2015 and 2030 under four different scenarios

		2015	Trend	Expansion	Sustainability	Conservation
Carbon pools	*c_above*	50.90	43.39	45.63	49.50	53.79
	*c_below*	15.22	14.71	15.57	15.66	15.88
	*c_soil*	506.07	487.16	494.15	503.57	516.31
	*c_dead*	14.29	12.04	12.71	13.87	15.16
LULC types	Cropland	100.75	100.96	100.97	101.03	100.56
	Forest	340.56	286.15	301.87	330.03	361.34
	Grassland	122.47	139.18	148.29	136.28	125.28
	Built-up land	12.71	30.95	16.31	13.95	13.95
	Unused land	9.98	0.06	0.62	1.30	0.01
Total		586.47	557.30	568.06	582.59	601.14
Terrestrial carbon storage change from 2015 to 2030		/	− 29.17	− 18.42	− 3.88	14.67

**Fig. 8:**
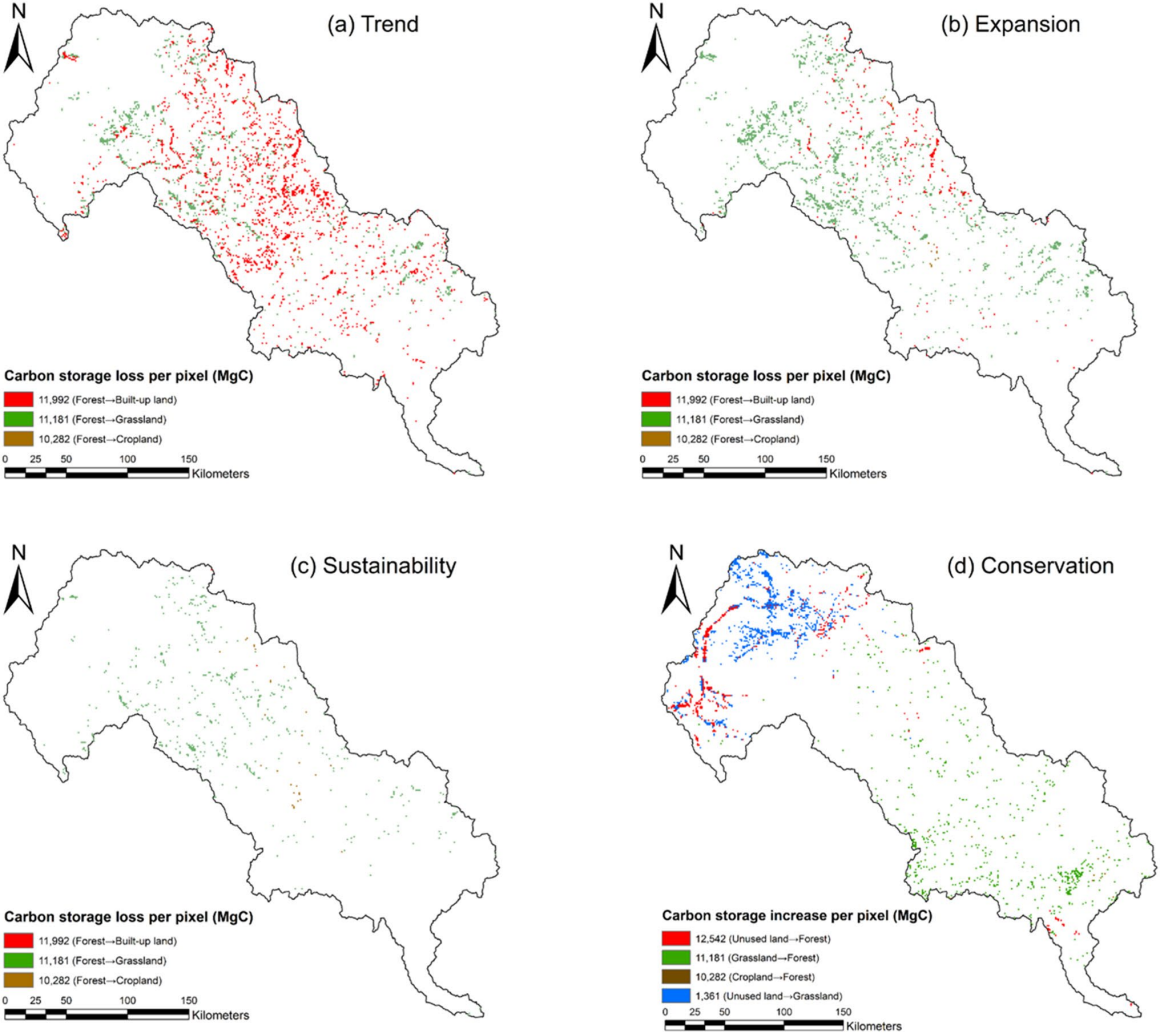
Terrestrial carbon storage change from 2015 to 2030 under (**a**) *Trend*, (**b**) *Expansion*, (**c**) *Sustainability* and (**d**) *Conservation* scenario in the LRB. For each pixel, the forests projected to be replaced by built-up lands would lead to the 11,991 MgC loss, the forests projected to be replaced by grasslands would lead to the 11,181 MgC loss, the forests projected to be replaced by croplands would lead to the 10,282 MgC loss, the unused lands projected to be replaced by forests would lead to the 12,542 MgC increase, the grasslands projected to be replaced by forests would lead to the 11,181 MgC increase, the croplands projected to be replaced by forests would lead to the 10,282 MgC increase, the unused lands projected to be replaced by grasslands would lead to the 1361 MgC increase

The loss of carbon stored in the forests is the main driver of the total terrestrial carbon storage loss from 2015 to 2030 in the LRB (Tables S1–S4, and Fig. 8). The carbon storage in the forests is projected to decrease from 340.6 MtC to 286.2 MtC under the *Trend* scenarios, 301.9 MtC under the *Expansion* scenarios, and 330.0 MtC under the *Sustainability* scenarios, which are all far greater than the increased carbon storage due to grassland growth. Under the *Trend*, *Expansion* and *Sustainability* scenarios, the terrestrial carbon storage loss in the LRB will occur due to the extensive forests projected to be replaced by built-up lands, grasslands and croplands (Fig. 7). The conversion from forest to built-up lands will mostly be responsible for terrestrial carbon storage loss under the *Trend* scenario, while the conversion from forests to the croplands is the main reason for terrestrial carbon storage loss under the *Expansion* and *Sustainability* scenarios. Meanwhile, the results from the modelling under *Conservation* scenario show the implementation of the ecological restoration and protection policy targets would significantly increase the amount of carbon stored in the forests and mildly increase the amount of carbon stored in the grassland, together with leading to the terrestrial carbon storage increase in the LRB (Fig. 8).

## Discussion

### Performance evaluation of the model

Generally, the CLUMondo model has high accuracy with an overall *Kappa simulation* of 0.86 (Table 8). *Kappa transition* is close to 1, indicating its robust ability to simulate the number of land system changes in the LRB. The *Kappa transition location* value is as high as 0.87, which indicates that the spatial allocation of these land system changes is much more accurate than random. Kappa values above 0.8 represent strong agreement or accuracy between two maps (Landis and Koch 1977). Therefore, the CLUMondo has good applicability in the LRB and can be used to predict the future simulation of land use change.

**Table 8:** Kappa simulation scores obtained from the assessment of the results of CLUMondo for 2015

Accuracy assessment index	Overall	Crop_ext	Crop_med	Crop_int	Forest	Grass_lls	Grass_hls	Water	Built-up	Unused land
Kappa simulation	0.86	0.85	0.89	0.85	0.91	0.87	0.94	0.81	0.41	0.78
Kappa transition location	0.87	0.86	0.90	0.90	0.92	0.87	0.94	0.83	0.41	0.85
Kappa transition	0.99	0.99	0.99	0.94	0.99	0.99	0.99	0.97	0.99	0.91

Moreover, the *Kappa simulation* values for all land systems except the built-up land system exceeded 0.77. The disagreements between the built-up land systems in 2000 and 2015 can be attributed to locational discrepancies with lower *Kappa transition location* of 0.41. The area of built-up land only covers 1.47% of LRB in 2000, but the built-up land represents the largest changed area among all land system types between 2000 and 2015, which increased the area cover to 3.44% of the LRB in 2015. Even though, the results showed that the land system changes were simulated with relatively high accuracy compared with the performance of previous similar regional-scale CLUMondo applications (Jin et al. 2019; Liu et al. 2017; van Vliet et al. 2011), of which the *Kappa simulation* values vary between 0.2 and 0.6.

### Terrestrial carbon storage and land system evolution: implications for the SDGs

The LRB is the most afforested river basin in North China (Yang et al. 2019). The forest in the LRB is a significant part of China’s Three-North Shelter Forest Program, also known as the “Green Great Wall” because its massive area spans half of northern China since the late 1970s. Our simulation demonstrates that the large areas of forests in the LRB continue to be the largest carbon storages in both the vegetation and the soil in the future. The average estimated carbon density in the LRB was 128.1 MgC ha^−1^ in 2015, which is 1.2 times China’s average of 107.1 MgC ha^−1^ (Xu et al. 2018a). Therefore, the LRB plays a significant role in storing and capturing carbon and mitigating carbon emissions. However, the simulation results showed that deforestation contributed to forest system change under all scenarios except *conservation* scenarios, and the loss of carbon stored in the forests is the main driver of the total terrestrial carbon storage loss from 2015 to 2030 in the LRB (Tables S1 to S4, and Fig. 8). Therefore, maintaining and protecting forest ecosystems is critical to mitigating climate change (SDG 13) (Krause and Tilker 2021).

Since 1990, it is estimated that 420 million hectares of forest have been lost globally (FAO and UNEP 2020). Over the past decade, there has been increasing international concern around deforestation and its impact on climate change (Mackey et al. 2015). The United Nations’ Sustainable Development Goals (adopted in 2016) aim to fully halt deforestation (UNDP 2015). Therefore, it is essential to take urgent action to combat climate change and its impacts by afforestation and sustainable forests management. As the LRB is the most afforested river basin in north China, it is particularly effective and efficient for the government to promote sustainable forest management, including climate change mitigation in the forest sector, by linking different policy measures. Such activities would provide co-benefits for both climate change mitigation (through increased carbon stocks) and adaptation by increasing ecosystems’ resilience to climate-related hazards and related disasters. Since 2015, a series of policies promoting afforestation have been implemented in the LRB, including; ‘National Forest Management Planning (2016–2050)’ (State Forestry Administration of China 2016), ‘Land greening planning of Hebei Province (2018–2035)’ (Hebei Provincial Department of Natural Resources 2018), and ‘Implementation plan of afforestation in Zhangjiakou city and Chengde Bashang area of Hebei Province’ (State Forestry Administration of China 2019). Such policies are encouraging from the perspective of climate change mitigation (SDG13), and could contribute to the formulation of more ambitious sustainable forest management policies that can be considered in the future.

In our simulations, most deforestation is projected to occur around urban areas and where grassland and cropland systems replace the forests. Therefore, in addition to forest protection, it would be possible to compensate for the carbon loss due to deforestation by increasing the carbon storage in the grassland and cropland systems and decreasing the carbon emission due to grazing and agricultural activities. Climate-smart agriculture (CSA) has been promoted as a systematic approach for developing agricultural strategies to ensure sustainable food security in the context of mitigation practices, promoting carbon sequestration from the atmosphere (Lipper et al. 2014; Palombi and Sessa 2013). In addition to advancing climate (SDG 13) and food security (SDG 2), when a CSA approach is well designed, it can also contribute to urban development (SDG11) and life on land (SDG 15) (FAO 2019). In the North China Plain, the CSA practices have been demonstrated to decrease the food carbon footprint and increase nitrogen use efficiency and irrigation water-use efficiency since 2000 (Xin and Tao 2021). Considering the topographical characteristics and meteorological environment of the LRB, the management practices of adding cover crops into the crop rotation (Kaye and Quemada 2017), applying biochar to soils (Hou et al. 2020), and minimising soil tillage (i.e. conservation tillage) (Zhen et al. 2020) could be promoted and incorporated into regional plans.

All scenarios indicate that the LRB is likely to face both land cover change and land use intensification in socio-economic development and environmental conservation between 2015 and 2030, while the change trajectories varied greatly under different scenarios in various parts of the LRB (Fig. S1). The southern, north-western and middle parts of the LRB are characterised by low topographic relief, intensive human activity, and mixed cropland and urban systems. China has set urbanisation as one of the core development strategies for economic growth and social development in its recent Five-Year plans (Cui et al. 2019). In the LRB, expanding the built-up system is projected to be around the original urban areas, expanding into the surrounding areas under all four future scenarios. Particularly, in the *Trend* and *Expansion* scenarios, extensive unused land near the urban areas would be utilised for built-up areas. However, not all of these urban expansions under future scenarios will lead to the strengthening of the sustainable planning and management of urbanisation in China, in the context of SDG 11 (sustainable cities and human settlements). Rapid urbanisation exerts pressure on food and fresh water supplies, the living environment, and public health. The conservation activities such as afforestation under the *Conservation* scenario would benefit the achievement of SDG 13 (climate change), although it could hinder the local economic development. Such comprehensive trade-offs and synergies between different SDGs under all future scenarios have been analysed based on the SDG Interlinkages Tool (Zhou et al. 2017) and showcased for the LRB by Zhou et al. (2021; submitted manuscript in this special feature). In this study, we will discuss the selected SDGs, which are most relevant to the LRB.

The demand for crop and livestock is expected to increase in the future (SDG 2). However, the cropland (i.e. crop_ext, crop_med, and crop_int) and grassland (i.e. grass_lls and grass_hls) cover is rather limited in the LRB. The traditional extensive farming pattern was replaced by cropland systems mainly through intensification due to the crop demand for rapid urbanisation in the LRB. The high demand for livestock in the future will result in the intensification of grazing activities in the grassland. The results show that the intensification is projected to be the main source of future grassland system change, widely distributed in the upper stream of LRB. As agriculture is the greatest water-use sector in China (Li et al. 2021), this projected agricultural intensification would be crucial to water scarcity (SDG 6) in the LRB. The future forests area in the LRB would also be relevant to future water quantity and quality (SDG 6), climate change (SDG 13), and life on land (SDG 15). Hence, it is of interest and necessity to discuss the interlinkages of SDG 2 (zero hunger), SDG 6 (water), SDG11 (sustainable cities), SDG 13 (climate change) and SDG 15 (life on land) in the context of LRB’s land system evolution under future scenarios at the sub-national scale.

### Trade-offs and synergies between SDG 2 (zero hunger), SDG 6 (water), SDG11 (sustainable cities), SDG 13 (climate change) and SDG 15 (life on land)

Globally, human water security, the health of aquatic environments and river biodiversity have been greatly impacted by climate change and human socio-economic development over the past few decades (Jacobsen et al. 2012; van Vliet et al. 2013; Vörösmarty et al. 2010). Water scarcity is an imbalance of water supply and demand. It refers to the relative shortage of water in a water supply system which can be affected by the supply, demand and quality of water in a river basin due to climate change or human actions (Liu et al. 2016; Pereira et al. 2009). The SDGs aim to address these issues, focusing on water and sanitation in SDG 6 and related targets in many other goals. The BTH region is the most severely region in China affected by water scarcity (Li et al. 2017a). Although the new South-to-North Water Transfer Project (Zhang 2009) could mitigate part of the water scarcity issue in the BTH, the LRB currently still plays an important water supply function for Tianjin city, a key metropolis of the BTH. The land system changes under future scenarios in the LRB indicate the challenge to achieve the SDG target 6.1 (drinking water for all), target 6.3 (water quality), target 6.4 (address water scarcity), and SDG11 (sustainable cities) in the sub-national regions such as BTH.

Water scarcity in the LRB is likely to increase in the near future. On the one hand, providing sufficient food, increasing productivity and production (SDG target 2.1 and 2.4), and substantially increasing the number of cities and human settlements (SDG target 11.b) will lead to an increase in the water demand in the LRB due to the growth of human population, crop cultivation and grazing, and rapidly changing diets, including greater consumption of animal source foods (Harris et al. 2020; Lehner et al. 2006; Rosegrant et al. 2009). However, due to rainfall reduction, land use change and construction of many small check dams for soil and water conservation, the average annual runoff had decreased by approximately 30% since the 1980s (Ping et al. 2008). By 2040, the sustainable water supply in Haihe River Basin (containing the LRB) will be more challenging due to the warmer and drier climate and more intense extreme weather events (Chu et al. 2010).

On the other hand, nonpoint-source pollution due to anthropogenic activities such as LULC is the main factor affecting surface water quality (Charalampous et al. 2015; Rong et al. 2009; Zhou et al. 2014), impacting upon the achievement of target 6.3 (water quality). All future scenarios indicate a significant increase in intensively managed cropland and grassland systems in the LRB. However, the intensive cropping practices (e.g. mechanisation) and improved nutrient management (e.g. high agrochemical inputs) for increasing crop and grass yield frequently will result in negative impacts on water quality, including runoff of sediments and agrochemicals to surface waters, as well as biodiversity loss and reductions in cultural services (Benayas and Bullock 2012; Ju et al. 2007; Tsiafouli et al. 2015). Also, the change of climatic conditions in the future may lead to some uncertainties in water environment quality improvement, risk prevention and control effectiveness (Michalak 2016). In particular, the projected increased intensity and frequency of extreme weather events in the LRB will increase the possibility of pollution incidents and make water environment risk prevention more difficult in the future. Overall, the results show that the LRB will suffer more from both quantity- and quality-induced water scarcity problems in the future, it is therefore of importance to address the changes in water scarcity under the effect of rapid urbanisation. Special attention to environmental management and sustainable land system design must be directed towards reducing water pollution and encouraging water conservation for minimising the trade-off between SDG 2 (zero hunger), SDG 6 (water), SDG 11 (sustainable cities), and maximising their synergies.

Due to forests' capacity to store and capture carbon (Katila et al. 2017; Popkin 2019; Seymour and Busch 2016), improve air quality (Eisenman et al. 2019; Nowak et al. 2006), soil and water conservation (Biao et al. 2010; Zhu et al. 2018) and maintain biodiversity (Sayer et al. 2019), forests dynamics and how human societies interact with forests have been associated with SDG 6 (water), SDG11 (sustainable cities), SDG 13 (climate change) and SDG 15 (life on land), and play a significant role in ecosystem services (Xu et al. 2021; submitted manuscript in this special feature) in the LRB. However, unsustainable urban expansion (i.e. *Trend* and *Expansion* scenarios) will significantly reduce carbon storage (SDG 13; Table 8), by replacing a large number of forests with built-up lands (SDG target 11.b), croplands and grasslands (SDG target 2.1 and 2.4) (Fig. 8). Such deforestation due to unsustainable urban expansion will also hinder the achievement of SDG target 6.3 (water quality), target 11.6 (air quality), and several targets of SDG 15 (i.e. targets 15.1, 15.2, 15.4, 15.5, 15.8). Compared with the economic expansion-oriented development plan, the sustainable shared socio-economic pathway (*Sustainability* scenario) and conservation practices (*Conservation* scenario) are likely to increase carbon uptake in the LRB’s terrestrial ecosystems and compensate for the carbon loss due to the socio-economic development and population growth in the future. Therefore, implementing future ecological restoration projects and protection policies could be an important strategy for maximising the synergy of SDGs 6, 11, 13, 15. However, under the *Conservation* scenario, several grassland systems with low-density livestock and extensive cropland systems with low crop production potential convert to forest in the northwest part of the LRB. In this case, the implementation of afforestation could hinder the achievement of SDG 2, though this may not necessarily be so, since the agricultural intensification and the CSA mentioned above could also achieve growth of food production.

### Limitations

In this study, even though, the results showed that the land system changes were simulated with relatively high accuracy compared with the performance of previous similar regional-scale CLUMondo applications (Jin et al. 2019; Liu et al. 2017; van Vliet et al. 2011), the generalisation performance of the prediction was not high for land systems with less area (e.g. built-up land) during the training period (2010–2015). This could lead to the underestimation or overestimation of the land system changes in the LRB to some extent. In addition, in the CLUMondo, all demands (i.e. crop production, livestock, urban land and forest areas) were assumed to maintain a steady annual percentage change, and agriculture production efficiency (i.e. crop and livestock) on different land systems were assumed unchanged from 2015 to 2030. In reality, changing environmental conditions, constraints on different land systems, and consumer behaviour will impact the region's trade balance and demand. Future climate change's uncertainty and agricultural advancements will also affect future agricultural production and agriculture productions' efficiency on different land systems.

It is often difficult to get consensus for designing the local scenario in a stakeholder meeting. There is a universal limitation for this approach in that the attitude of each individual stakeholder towards the future is undeniably dependent on a stakeholder’ own knowledge, the experience of the surrounding environment, as well as the stakeholder’ implicit assumptions about the future (Hewitt et al. 2014; Milestad et al. 2014; Patel et al. 2007). For, minimising this discordance, we used the focus group discussion approach to summarise the stakeholders’ ideas in the stakeholder meeting, and to finalise the scenarios which are scientifically sound and most acceptable for the stakeholders based on the integrated methodology considering the different socio-economic development and environmental protection targets, local plans and policies, and the information shared from the stakeholders’ workshop.

Another limitation is due to the uncertainty and emerging knowledge after the scenarios for this study were developed. China recently announced the climate goal to achieve carbon neutrality before 2060, the rapid introduction of renewable energy for decarbonisation and potential for social–technological innovations for the carbon neutrality target could also impact future land systems change (Bowyer and Kretschmer 2010; Poggi et al. 2018; van de Ven et al. 2021; Xiao et al. 2021), and the achievement of SDGs, such as SDG 9: Industry, innovation, and infrastructure and SDG 17: Partnership for the goals (Hinson et al. 2019; Sinha et al. 2020; Walsh et al. 2020). Nevertheless, these effects have not been fully considered by the SSP scenarios used in this study. This study only simulates the future land use change until 2030 to align with the SDG targets achievement date, but future research could generate new knowledge and the an amended SSP for China or even the LRB to better understand the prospective role of land use in achieving the SDGs and contributing to a carbon-neutral China by 2060.

Furthermore, the COVID-19 pandemic will also impact the development of pathways from different scenarios. The COVID-19 pandemic has brought an unprecedented threat to public health in all countries and the global economy. Although the sustainable recovery from COVID-19 in China should be taken into account in China’s 14th Five-Year Plan (Ahmad et al. 2020), COVID-19 could threaten the achievement of some of the SDGs, such as SDG 6: access to water and sanitation (The Lancet Public 2020), and SDG 2: food security (Laborde et al. 2020). Therefore, the scenarios presented should be seen as experiments of what is feasible in terms of meeting such demands (i.e. crop production, livestock, urban land and forest areas) under various constraints.

It should be noted that the methodology of the InVEST carbon model relies on the differences in carbon densities between LULC types. In this study, we have used the available data of carbon pools that fit with the study area to the greatest extent possible. However, for some carbon pools where measured data for the LRB or Hebei province are not available, we used data derived from the national mean carbon densities in China. We believe this would only have a negligible impact on the total terrestrial carbon storage of the LRB since the carbon pools of forest and grassland derived from the in situ-measured data account for the overwhelming majority (more than 75%) of terrestrial carbon storage of the LRB. In future studies, additional in situ-measured carbon pool data would be helpful for modelling the carbon storage more reliably. Besides, the InVEST assumes that none of the LULC types in the landscape is gaining or losing carbon over time. The use of average inventory values for carbon fails to account for variation within a LULC type due to many factors, including land management history, temperature or elevation (Chaplin-Kramer et al. 2015). However, the carbon densities in biomass and soil can be influenced by many factors and will change during the modelling period. For example, the change of tillage practices and land management on cropland in the future will impact the soil organic carbon stability and density of cropland (Di et al. 2017; Luo et al. 2019; Zhao et al. 2015). Also, the carbon density of biomass in forests should increase over time, which means the carbon loss due to the future deforestation of aged trees will be significantly larger than the afforestation-leading carbon gain in the biomass (Fang et al. 2007, 2014). Another limitation is the carbon dynamic from one pool to another is not captured in the model. For example, if trees in a forest die due to disease, much of the carbon stored in aboveground biomass becomes carbon stored in other (dead) organic material. Even so, due to a lack of appropriate process-based models, this space for time substitution models which do not require site-specific calibration, such as InVEST is currently necessary (Sharps et al. 2017).

## Conclusions

Through the use of land system modelling, we investigated some potential trade-offs and synergies between different SDGs under different socio-economic and environmental targets. LULC change is closely related to the sustainable development of a region and local planning and policy. The land systems in the LRB are not only essential elements of any strategy to stabilise our climate (e.g. forests for SDG 13: climate action) as the natural means of carbon capture and storage, but also they are playing important roles in food provisioning (e.g. cropland and grassland for SDG 2: zero hunger), water security (SDG 6: water and sanitation), urbanisation (SDG11: sustainable cities), and biological diversity (SDG 15: life on land). Therefore, the LRB presents an optimum case for analysing SDGs' trade-offs and synergies at the sub-national scale.

In this study, the land system approach, which provides comprehensive information on socio-ecological factors, was used to explore the potential land system changes of the LRB by 2030. Four scenarios, which considered multiple demands for commodities and services, representing different pathways of managing LRB’s land resources, were simulated using the CLUMondo model. The simulated land use maps are freely available from NERC Environmental Information Data Centre (10.5285/a94640dc-fe21-4c38-936b-d62dfca0c952). The intensification of land systems has been acknowledged as a significant adaptation to population growth (Butsic and Kuemmerle 2015; Kuemmerle et al. 2013). All future scenarios indicate a significant increase in intensively managed cropland and grassland systems (SDG 2) and urban growth (SDG 11) in the LRB, leading to increased water scarcity (SDG 6). The unused land needs to be exploited to meet the increased demand for livestock, cropland, urban area and forest in different scenarios. Apart from the *Conservation* scenario, the forest areas (SDG 15) are projected to decrease under three scenarios by 2030, resulting in decreased carbon storage (SDG 13) in the LRB. To minimise the trade-offs and maximise the synergies between urbanisation and environmental and biodiversity conservation, the posed additional challenges to the region's scarcer land is unavoidable (Eitelberg et al. 2016; Van Asselen and Verburg 2013). The LRB needs policy coherence and synergies, with the integrated thinking of placing the nexus at the centre in meeting the sustainable demands across the water, energy, food, and biodiversity sectors. The LRB needs to embrace greater participation and transparency to optimise important policy gains in effectiveness.

Our findings will help understand future land use patterns under various social demands in the LRB but could have important development implications beyond the LRB. Potential trade-offs between economic development and environment protection exist in many sub-national scale large river basins in China and beyond. Our findings can guide regional sustainable development and rational utilisation of land resources in other sub-national scale river basins in China, and will be valuable for policy and planning purposes to the pursuance of SDGs at the whole national scale. Furthermore, the results can be used in further modelling of SDG synergies and trade-offs, for example, the results from this study were used to provide quantitative and qualitative information to the SDG interlinkages tool (https://sdginterlinkages.iges.jp/luanhe/index.html) (Zhou et al. 2021; submitted manuscript in this special feature).

## Supplementary Information

Below is the link to the electronic supplementary material.

Supplementary file1 (DOCX 626 KB)
