# Supplementary material for: Modelling land system evolution and dynamics of terrestrial carbon stocks in the Luanhe River Basin, China: a scenario analysis of trade-offs and synergies between sustainable development goals
Source: Sustain Sci. 2021 Jul 16;17(4):1323–45. doi: 10.1007/s11625-021-01004-y (PMC8282888; doi:10.1007/s11625-021-01004-y)
Supplement: Supplementary file 1 — Supplementary file1 (DOCX 626 KB) [file 11625_2021_1004_MOESM1_ESM.docx]

*Sustainability Science*

Supporting Information for

**Modelling land system evolution and dynamics of terrestrial carbon stocks in the Luanhe River Basin, China: a scenario analysis of trade-offs and synergies between Sustainable Development Goals**

Jiren Xu^1^, Fabrice G. Renaud^1^, Brian Barrett^2^

^1^ School of Interdisciplinary Studies, University of Glasgow, Dumfries, DG1 4ZL UK

^2^ School of Geographical & Earth Sciences, University of Glasgow, Glasgow, G12 8QQ UK

^*^ Corresponding author. Email address: Jiren.Xu@glasgow.ac.uk; jiren.xu@hotmail.com.

Tel: +44 (0)1387702091

**Contents of this file**

Table S1 to S4, Figure S1

**Introduction**

This file of supplemental material contains four tables and one figure. Table S1 shows cross-tabulation tables of land system class changes between 2015 and 2030 under *Trend* scenario. Table S2 shows cross-tabulation tables of land system class changes between 2015 and 2030 under *Expansion* scenario. Table S3 shows cross-tabulation tables of land system class changes between 2015 and 2030 under *Sustainability* scenario. Table S4 shows cross-tabulation tables of land system class changes between 2015 and 2030 under *Conservation* scenario. Figure S1 shows the spatial distribution of land system changes in the LRB under the four scenarios.

**Table S1** Cross-tabulation tables of land system class changes between 2015 and 2030 under *Trend* scenario (unit: km^2^)

| **2030**  **2015** | **Extensive cropland** | **Medium intensive cropland** | **Intensive cropland** | **Forest** | **Grassland with low livestock** | **Grassland with high livestock** | **Water** | **Built-up area** | **Unused land** |
| --- | --- | --- | --- | --- | --- | --- | --- | --- | --- |
| **Extensive cropland** | 2929 | 2973 | 145 | 93 | 167 | 85 | 22 | 580 | 1 |
| **Medium intensive cropland** | 0 | 2240 | 491 | 9 | 22 | 11 | 21 | 141 | 0 |
| **Intensive cropland** | 0 | 0 | 593 | 0 | 2 | 2 | 3 | 59 | 0 |
| **Forest** | 133 | 207 | 22 | 13717 | 1358 | 137 | 31 | 1736 | 0 |
| **Grassland with low livestock** | 100 | 80 | 14 | 529 | 10555 | 2222 | 23 | 314 | 1 |
| **Grassland with high livestock** | 7 | 2 | 0 | 0 | 1 | 488 | 17 | 2 | 0 |
| **Water** | 11 | 33 | 26 | 8 | 181 | 9 | 424 | 19 | 1 |
| **Built-up area** | 85 | 379 | 131 | 99 | 127 | 68 | 21 | 724 | 0 |
| **Unused land** | 8 | 2 | 5 | 3 | 723 | 5 | 0 | 392 | 5 |

**Table S2** Cross-tabulation tables of land system class changes between 2015 and 2030 under *Expansion* scenario (unit: km^2^)

| **2030**  **2015** | **Extensive cropland** | **Medium intensive cropland** | **Intensive cropland** | **Forest** | **Grassland with low livestock** | **Grassland with high livestock** | **Water** | **Built-up area** | **Unused land** |
| --- | --- | --- | --- | --- | --- | --- | --- | --- | --- |
| **Extensive cropland** | 3878 | 2107 | 62 | 98 | 172 | 100 | 19 | 557 | 2 |
| **Medium intensive cropland** | 0 | 2287 | 444 | 9 | 19 | 15 | 21 | 140 | 0 |
| **Intensive cropland** | 0 | 0 | 593 | 0 | 4 | 2 | 3 | 57 | 0 |
| **Forest** | 188 | 157 | 19 | 14473 | 1999 | 185 | 30 | 290 | 0 |
| **Grassland with low livestock** | 120 | 60 | 14 | 551 | 9747 | 3114 | 22 | 184 | 26 |
| **Grassland with high livestock** | 7 | 2 | 0 | 0 | 3 | 488 | 17 | 0 | 0 |
| **Water** | 16 | 27 | 26 | 9 | 229 | 11 | 376 | 17 | 1 |
| **Built-up area** | 134 | 335 | 126 | 109 | 112 | 87 | 20 | 711 | 0 |
| **Unused land** | 10 | 0 | 5 | 3 | 926 | 8 | 0 | 134 | 57 |

**Table S3** Cross-tabulation tables of land system class changes between 2015 and 2030 under *Sustainability* scenario (unit: km^2^)

| **2030**  **2015** | **Extensive cropland** | **Medium intensive cropland** | **Intensive cropland** | **Forest** | **Grassland with low livestock** | **Grassland with high livestock** | **Water** | **Built-up area** | **Unused land** |
| --- | --- | --- | --- | --- | --- | --- | --- | --- | --- |
| **Extensive cropland** | 4794 | 1199 | 54 | 103 | 228 | 30 | 24 | 556 | 7 |
| **Medium intensive cropland** | 0 | 2296 | 435 | 10 | 26 | 6 | 23 | 139 | 0 |
| **Intensive cropland** | 0 | 0 | 593 | 0 | 5 | 0 | 3 | 56 | 2 |
| **Forest** | 240 | 115 | 18 | 15824 | 1067 | 16 | 31 | 30 | 0 |
| **Grassland with low livestock** | 135 | 46 | 13 | 604 | 12506 | 279 | 24 | 197 | 34 |
| **Grassland with high livestock** | 7 | 2 | 0 | 0 | 3 | 488 | 17 | 0 | 0 |
| **Water** | 23 | 18 | 26 | 10 | 80 | 0 | 537 | 16 | 2 |
| **Built-up area** | 164 | 306 | 125 | 120 | 179 | 11 | 23 | 705 | 1 |
| **Unused land** | 10 | 0 | 5 | 4 | 901 | 1 | 0 | 89 | 133 |

**Table S4** Cross-tabulation tables of land system class changes between 2015 and 2030 under *Conservation* scenario (unit: km^2^)

| **2030**  **2015** | **Extensive cropland** | **Medium intensive cropland** | **Intensive cropland** | **Forest** | **Grassland with low livestock** | **Grassland with high livestock** | **Water** | **Built-up area** | **Unused land** |
| --- | --- | --- | --- | --- | --- | --- | --- | --- | --- |
| **Extensive cropland** | 4725 | 1245 | 54 | 140 | 208 | 57 | 22 | 543 | 1 |
| **Medium intensive cropland** | 0 | 2295 | 437 | 16 | 25 | 5 | 21 | 136 | 0 |
| **Intensive cropland** | 0 | 0 | 593 | 15 | 2 | 2 | 3 | 44 | 0 |
| **Forest** | 223 | 105 | 18 | 16337 | 530 | 67 | 31 | 30 | 0 |
| **Grassland with low livestock** | 134 | 46 | 13 | 1268 | 11161 | 944 | 23 | 249 | 0 |
| **Grassland with high livestock** | 7 | 2 | 0 | 7 | 3 | 481 | 17 | 0 | 0 |
| **Water** | 22 | 18 | 26 | 16 | 142 | 4 | 466 | 18 | 0 |
| **Built-up area** | 164 | 307 | 125 | 149 | 127 | 33 | 22 | 707 | 0 |
| **Unused land** | 10 | 0 | 5 | 308 | 757 | 1 | 0 | 61 | 1 |


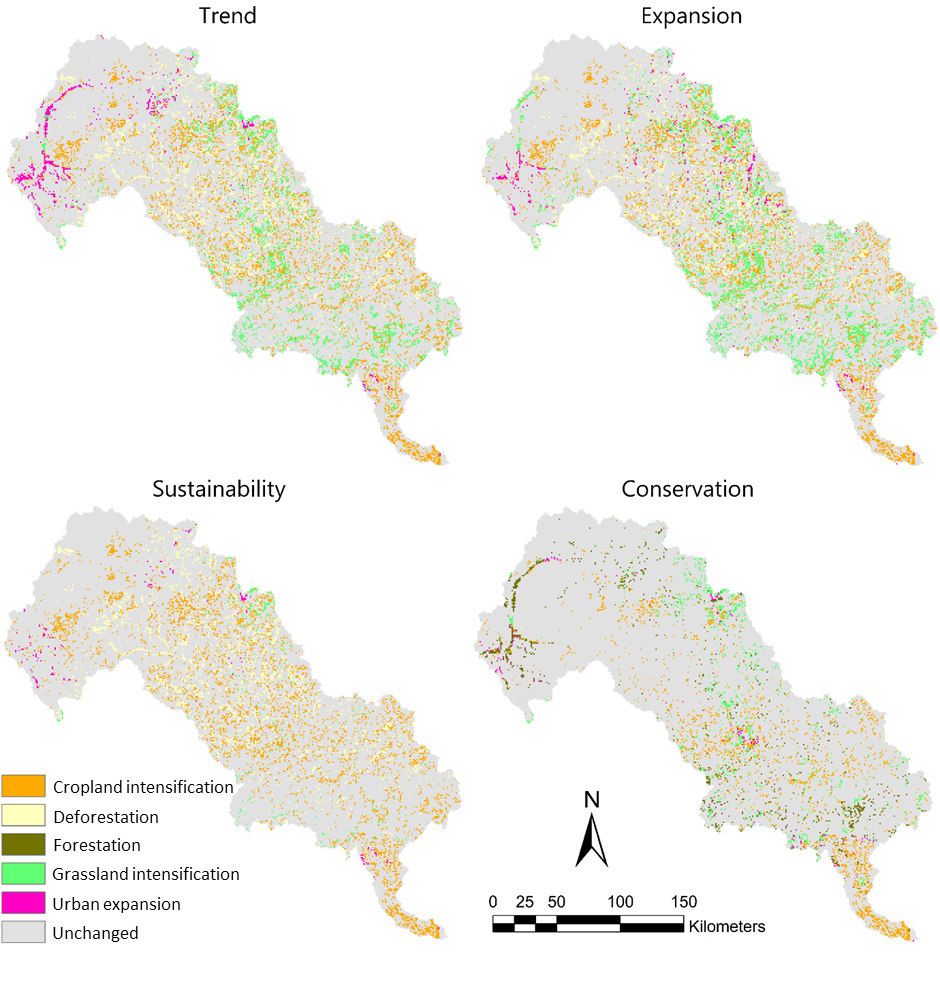


**Fig. S1.** Spatial distribution of land system changes in the LRB under the four scenarios.
